# Proteomic signatures of serum albumin-bound proteins from stroke patients with and without endovascular closure of PFO are significantly different and suggest a novel mechanism for cholesterol efflux

**DOI:** 10.1186/1559-0275-12-2

**Published:** 2015-01-13

**Authors:** Mary F Lopez, Bryan Krastins, David A Sarracino, Gregory Byram, Maryann S Vogelsang, Amol Prakash, Scott Peterman, Shadab Ahmad, Gouri Vadali, Wenjun Deng, Ignacio Inglessis, Tom Wickham, Kathleen Feeney, G William Dec, Igor Palacios, Ferdinando S Buonanno, Eng H Lo, MingMing Ning

**Affiliations:** Thermo Scientific BRIMS, 790 Memorial Dr, Cambridge, MA 02139 UK; Clinical Proteomics Research Center and Cardio-Neurology Clinic, Department of Neurology, Massachusetts General Hospital, Harvard Medical School, Boston, MA USA

**Keywords:** Biomarker, Discovery, Stroke, Cerebrovascular disease, Ischemic stroke, Patent foramen ovale, PFO, Mass spectrometry, Proteomics, Albumin

## Abstract

**Background:**

The anatomy of PFO suggests that it can allow thrombi and potentially harmful circulatory factors to travel directly from the venous to the arterial circulation – altering circulatory phenotype. Our previous publication using high-resolution LC-MS/MS to profile protein and peptide expression patterns in plasma showed that albumin was relatively increased in donor samples from PFO-related than other types of ischemic strokes. Since albumin binds a host of molecules and acts as a carrier for lipoproteins, small molecules and drugs, we decided to investigate the albumin-bound proteins (in a similar sample cohort) in an effort to unravel biological changes and potentially discover biomarkers related to PFO-related stroke and PFO endovascular closure.

**Methods:**

The method used in this study combined albumin immuno-enrichment with high resolution LC-MS in order to specifically capture and quantify the albumin-bound proteins. Subsequently, we measured cholesterol and HDL in a larger, separate cohort of PFO stroke patients, pre and post closure.

**Results:**

The results demonstrated that a number of proteins were specifically associated with albumin in samples with and without endovascular closure of the PFO, and that the protein profiles were very different. Eight proteins, typically associated with HDL were common to both sample sets and quantitatively differently abundant. Pathway analysis of the MS results suggested that enhanced cholesterol efflux and reduced lipid oxidation were associated with PFO closure. Measurement of total cholesterol and HDL in a larger cohort of PFO closure samples using a colorimetric assay was consistent with the proteomic predictions.

**Conclusions:**

The collective data presented in this study demonstrate that analysis of albumin-bound proteins could provide a valuable tool for biomarker discovery on the effects of PFO endovascular closure. In addition, the results suggest that PFO endovascular closure can potentially have effects on HDL, cholesterol and albumin-bound ApoA-I abundance, therefore possibly providing benefits in cardioprotective functions.

**Electronic supplementary material:**

The online version of this article (doi:10.1186/1559-0275-12-2) contains supplementary material, which is available to authorized users.

## Introduction

Patent Foramen Ovale (PFO), a congenital cardiac abnormality where the left and right atria of the heart are connected, is highly prevalent (25%-30%) in the general population [1]. PFO’s caused more than 250,000 strokes in the United States, allowing peripheral embolisms to travel directly to the brain and are also associated with debilitating migraine headaches [2]. Emerging studies show that PFO-related neurovascular disease is a multi-organ condition with varying individual risk factors that may require individualized therapeutic approaches - opening the field for new pharmacologic and therapeutic targets such as PFO endovascular closure [1].

Although in asymptomatic patients PFO is not necessarily considered a disease, the full effects of this congenital abnormality on health are not clear. Clinical trials to investigate treatment options for ischemic stroke, including PFO endovascular closure are ongoing, but because individual risks vary, and treatments may need to be tailored to each individual patient, controversies regarding PFO and PFO closure persist. In part, we believe this is due to a poor understanding of the molecular landscape of PFO-related neurovascular injury. Endovascular closure of PFO provides a rare bedside model in which to study the effects of a specific mechanical intervention on circulatory protein signaling, both immediately and over time [3, 4].

Clinical proteomics is an ideal and promising approach for uncovering the changes in complex multi-organ diseases in PFO-related disease since PFO allows for unknown, but potentially harmful and vasoactive mediators to travel directly to the brain triggering stroke, unrelenting migraine and altering circulatory phenotype in blood [3].

Our previous publication using high-resolution LC-MS/MS to profile protein expression patterns in plasma demonstrated a collection of differently abundant proteins, including albumin and ApoA1, before and 12 months after stroke related PFO closure [4]. In a recent publication investigating the association of serum albumin concentrations and ischemic strokes [5], the authors found a significant association between low serum albumin levels and, in particular cardioembolic and cryptogenic infarctions suggesting PFO might be a factor.

It is well known that albumin has pleiotropic functions including maintenance of plasma oncotic pressure, binding a host of toxic molecules and acting as a carrier for lipoproteins, small molecules and drugs [6–10]. Many previous publications have proposed that low abundance biomarkers for many diseases may be associated with albumin since binding to albumin extends the half-life of smaller proteins and protein fragments that may otherwise be rapidly cleared by the kidneys [11–15]. In fact, the likelihood that key biomarkers may exist in blood mainly bound to albumin has potentially confounded the success of many plasma or serum-based biomarker discovery studies, since albumin and other abundant protein depletion strategies have been implemented in an effort to reduce the large dynamic range of these fluids [16].

Although ApoAI (the major component of HDL) and HDL are the well documented mediators of cholesterol efflux [17–19], recent studies have shown that albumin also mediates cholesterol efflux from cultured fibroblasts and endothelial cells [20–22] but the mechanism is not known.

Albumin is also actively being tested in clinical trials as a treatment for acute brain injury [23, 24]. In light of these and our prior findings, characterization of albumin-bound proteins with respect to physiological changes could potentially be paradigm changing.

In the current study, we present data suggesting that albumin-bound proteins may be related to biological and physiological changes associated with PFO closure. To demonstrate this, we returned to the same sample set from the previous study and applied immunoenrichment coupled to mass spectrometry to capture and quantify albumin-bound proteins at baseline and 3–12 month follow up after stroke–related PFO, with and without endovascular closure [4]. Subsequently, we measured cholesterol and HDL in a larger, separate cohort of PFO stroke patients, pre and post closure. Based on our proteomic findings and pathway analysis predictions, we wanted to investigate if albumin-bound proteomic profiles were correlated to plasma HDL and cholesterol levels.

## Methods

### Clinical serum samples

Plasma samples were obtained from PFO stroke patients and healthy controls with similar risk factors from the Cardio-Neurology Clinic of Massachusetts General Hospital in accordance with IRB approval. PFO related “cryptogenic ischemic strokes” were identified by two vascular board certified neurologists. Rigorous inclusion/exclusion criteria were applied to ensure proper diagnosis in each group, and all patients underwent the following testing to rule out other etiology related to ischemic embolic infarct: 1) physical exam by a vascular neurologist to document clinical syndrome consistent with ischemic infarct; 2) MRI/MRA and CTA to document ischemic infarct and to rule out other reasons of infarct such as intracranial stenosis, large vessel occlusion, dissection; 3) transthoracic and/or transesophageal echocardiogram to assess and document the presence of PFO and rule out atrial/ventricular thrombus or any valvular lesions that may be related to embolism; 4) extended cardiac monitoring to rule out atrial fibrillation or other cardiac arrhythmia. In addition other ischemic stroke subtypes such as lacunar infarct related to hypertension and vasculitis, endocarditis and venous infarction were excluded. All patients with active infection, active pregnancy, or renal/liver failure which may alter protein signaling profiles and those with absence of baseline imaging such as CT or MRI needed to confirm clinical stroke syndromes were excluded. Controls were recruited from subjects with similar baseline risk factors to match the study population. Subject demographics are listed in Table 1. Patients were consecutively, prospectively enrolled post ischemic stroke in accordance with the approval of institutional IRB.

**Table 1:** **PFO stroke patient characteristics in separate validation cohort (n = 104)**

Patient characteristics (n = 104)		
Age	51.09 ± 12.02	
Male	55 (52.88%)	
White	89 (85.58%)	
Diabetes	9 (8.65%)	
Hypertension	41 (39.42%)	
Coronary artery disease	7 (6.73%)	
Migraine	31 (29.81%)	
Current smoker	17 (16.35%)	
Alcohol	43 (41.35%)	
History of stroke/TIA	39 (37.50%)	
Hyperlipidemia	32 (30.77%)	
Lipid_Lowering_Agent	71 (68.27%)	
	Pre-closure 60 (57.69%)	Post-closure 69 (66.35%)

All patients had CLIO certified cholesterol and HDL measured pre and post PFO closure at the same time interval as the exploratory cohort.

Plasma was obtained in EDTA coated tubes, immediately processed, aliquoted and frozen within 30 minutes of collection at −80°C to ensure minimal protein degradation. Standard operating procedures were strictly observed for all samples. All personnel were trained with SOP to process samples in the same fashion as follows: blood was obtained from venous source and immediately processed (within 5 min) to obtain plasma by centrifugation at 3400 RPM for 15 minutes at 20°C (to avoid platelet activation), removing the plasma supernatant without disturbing the clot, aliquoted immediately, and frozen at −80°C.

Samples were carefully transported frozen to be processed at the same time, in random order, by investigators blinded to the clinical data to avoid bias and batch variations.

### MSIA immuno-enrichment of albumin-bound proteins

**Figure 1:**
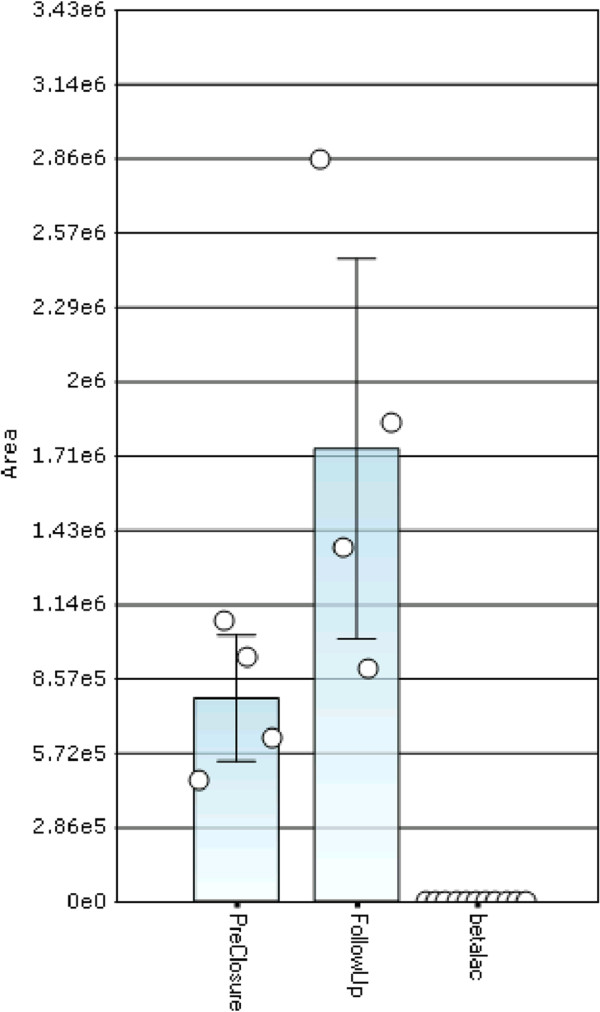
**Specificity of anti-albumin MSIA enrichment: Binding characteristics of peptide VQH[Oxid]LGA[A_42]PVT[Phosphoryl]LRAS[Phosphoryl]YLEIYNEQVR from Kinesin-like protein to MSIA micro columns activated with either anti-albumin or anti-beta lactoglobulin, antibodies.**

Custom MSIA tips activated with polyclonal anti-albumin or anti-betalactoglobulin antibodies were obtained from Thermo Fisher Scientific. All samples were processed with both anti-albumin and anti-betalactoglobulin tips using a Versette Automated Liquid Handler (Thermo Fisher Scientific) as previously described [25]. Specifically, the MSIA-Tips were mounted onto the head of the Versette and initially rinsed with assay buffer (10 mM PBS w/0.1%TWEEN 20), with 20 cycles (1 cycle consisting of a single aspiration and dispense of a 100 uL volume), from a single 200 uL buffer aliquot placed in the well of a microplate. Next, the MSIA-Tips were immersed into the wells of the microplate containing the samples, and 100 aspirations and dispense cycles were performed (50 uL volumes each), allowing for affinity capture of albumin and albumin-bound proteins. The MSIA-Tips were then rinsed with assay buffer (25 cycles) from another microplate, and 3 times with water (20 cycles each) from 3 more microplates (100 uL volumes aspiration and dispenses, from 200 uL placed in each well). The captured albumin and albumin-bound proteins were eluted with 33% acetonitrile/0.4% (v/v) formic acid by aspirating and dispensing a 20 uL volume 200 times, from a total of 50 uL in the wells of a microplate. The albumin and albumin-bound protein-containing eluates were then dried down in a SpeedVac concentrator until dry. Sufficient plasma (1ul or 50–80 ug) was processed to achieve saturation of the antibody bound to the MSIA tip resulting in normalization of the albumin concentration across all samples, and allowing the quantitative comparison of any proteins specifically associated with albumin. The anti-betalactoglobulin activated tips were included to control for any non-specific binding to the anti-albumin antibody since betalactoglobulin is not present in human plasma (Figure 1).

### Preparation of tryptic peptides for MS analysis

After MSIA processing, samples were eluted into a 96 well microplate, lyophilized and processed as previously described [24]. Briefly, the samples were rehydrated with 25 uL 4 M Urea/300 mM Tris/2.5% n-propanol/10 mM DTT. Subsequently, 2 uL 500 mM IAA in 1 M NH4CO3 was added. Next, 37.5 uL of 50 mM Tris/5 mM CaCl2 pH 8.0 and 12.5uL of 25 mM Acetic acid/trypsin (20 ug trypsin + 2.5 mL acetic acid) (Thermo Fisher Scientific). Samples were incubated at 37°C for 16 h. After digestion, each sample well was quenched with 3 uL of quench buffer ((333.3 fmol/ul PRTK (Pierce Retention Time Kit)/26.7% Formic Acid/H2O (Thermo Fisher Scientific), final volume 80 uL)). Twenty uL of each sample were subsequently injected into the mass spectrometer.

### Liquid chromatography and high-resolution mass spectrometry

Samples were prepared as described above and injected onto a Thermo Scientific Easy nLC system configured with a 10 cm × 100 um trap column and a 25 cm × 100 um ID resolving column. Buffer A was 98% water, 2% methanol, and 0.2% formic acid. Buffer B was 10% water, 10% isopropanol, 80% acetonitrile, and 0.2% formic acid. Samples were loaded at 4 uL/min for 10 min, and a gradient from 0-45% B at 375 nL/min was run over 130 min, for a total run time of 150 min (including regeneration and sample loading). The Thermo Scientific LTQ Orbitrap Velos mass spectrometer was run in a standard Top-10 data-dependent configuration, except that a higher trigger-threshold (20 K) was used to ensure that the MS2 did not interfere with the full-scan duty cycle. This ensured optimal full-scan data for quantification. MS2 fragmentation and analysis were performed in the ion trap mass analyzer.

### MS data analysis

Protein identification was performed using Thermo Scientific Proteome Discoverer version 1.4 (including Sequest and Percolator algorithms). The sequence database used for the searches was the 040212-RefSeqHuman.fasta. The Percolator peptide confidence filter was set to “high”. All Proteome Discoverer search parameters are provided in Additional file 1 and all identified proteins and peptide sequences are given in Additional file 2. Protein quantification was performed using Pinpoint version 1.4 software. The Pinpoint quantification workflow included importing the Proteome Discoverer .msf files as spectral libraries. Identified peptides were subsequently quantified in MS .raw files using the Pinpoint peak finding, chromatographic alignment and area calculation algorithms. Quantified proteins with all related information are given in Additional file 3. The LC-MS workflow precision was calculated (within Pinpoint) by quantification of the PRTC peptides across all samples yielding and average CV of 15%. Based on the precision calculation, differential abundance ratio limits for proteins were set to >1.2 or <0.8.

### Ingenuity pathways (IPA) data analysis

The differential expression results were uploaded and analyzed using IPA Downstream Effects (Ingenuity© Systems, http://www.ingenuity.com) according to the manufacturer’s instructions. IPA Downstream Effects attempts to identify functions that are expected to increase or decrease, given the observed gene or protein expression changes in the experimental dataset. Downstream Effects Analysis is based on expected causal effects between genes or proteins and functions; the expected causal effects are derived from the literature compiled in the Ingenuity® Knowledge Base. The analysis examines genes in the dataset that are known to affect functions, compares the genes’ direction of change to expectations derived from the literature, and then issues a prediction for each function based on the direction of change in the experimental samples relative to a control. If the direction of change is consistent with the literature across most genes, IPA predicts that the function will increase in the experimental sample. If the direction of change is inconsistent with the literature, IPA predicts that the function will decrease in the experimental sample and if the direction of change is not clear (there is no clear pattern related to the literature), IPA does not make a prediction. IPA uses the z-score algorithm to make predictions. The z-score algorithm is designed to reduce the chance that random data will generate significant predictions. The z-score predicts the direction of change for the function. An absolute z-score of ≥ 2 is considered significant. A biological function is: increased if the z-score is ≥ 2 and decreased if the z-score ≤ -2.

### Measurement of cholesterol and HDL

Cholesterol, triglycerides and HDL were measured colorimetrically on the Cobas c501 chemistry analyzer using the manufacturer’s recommended procedure (Roche Diagnostics).

## Results

### Differential abundance of albumin-bound proteins in baseline (B) *versus*3*–*12 month follow up (FU) samples

Differential abundance of the albumin-bound proteins was measured in samples from donors with and without PFO closure at baseline and at 3–12 month follow-up (Table 2). The experimental plan was as follows:

**Table 2:** **Donor sample set processed with immuno-enrichment and MS for identification and analysis of albumin-bound proteins**

Type of sample	Time point	n
PFO Closure	Baseline	16
PFO Closure	3-12 month Follow Up	5
PFO No Closure	Baseline	5
PFO No Closure	3-12 month Follow Up	5

1. To compare albumin-bound proteins from samples taken at baseline (B) and at 3–12 month follow up (FU). We chose to measure the abundance change over time as a reflection of the effects of PFO closure on protein profile during stroke recovery.
2. To obtain protein abundance ratios and ROC scores.
3. To compare results from PFO closure and no-closure samples.

Albumin coverage was approximately 86% (Additional file 2). Specificity for albumin-binding was assumed when binding to the anti-BL Ab was less than binding to the anti-albumin Ab (ratio of BL to albumin <0.5). In all measurements, the ratio of albumin itself at baseline (B) and follow up (FU) samples was *ca* 1 due to the intentional saturation of the antibody with sufficient plasma (see Methods), allowing an accurate measurement of the differential abundance of proteins specifically bound to albumin. As described in the methods, the LC-MS workflow precision was calculated using spiked-in (every sample) heavy isotope labeled peptides and the average CV was 15%. Based on the precision calculation, differential abundance ratio limits for proteins were set to >1.2 or <0.8.

**Figure 2:**
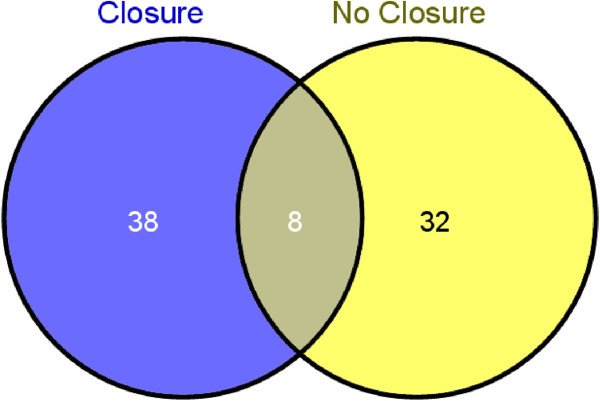
**Distribution of albumin-bound proteins in PFO closure** ***vs*** **no-closure sample sets.** Specificity for albumin-binding was assumed when binding to the negative control (anti-BL Ab) was less than binding to the anti-albumin Ab (ratio <0.5). Eight proteins were present in both sample sets and their abundance ratios and ROC AUC scores are given in Table 3. All 8 proteins present in both sample sets have been previously identified in HDL (26).

Additional file 4 lists all the albumin-bound proteins and abundance ratios in PFO closure and no-closure samples. A total of 46 albumin-bound proteins were detected in PFO closure samples, 15 with an increased ratio (>1.2) and 13 with a decreased ratio (<0.8). Forty albumin-bound proteins were detected in the PFO no-closure samples, 9 with an increased ratio (>1.2) and 19 with a decreased ratio (<0.8). Eight proteins were present in both sample sets (Figure 2), and their abundance ratios and ROC AUC scores are given in Table 3. All 8 proteins present in both sample sets have been previously identified in HDL [26]. The ROC AUC scores and abundance ratios of the 8 proteins in common were very different between the PFO closure and no closure sample groups. ApoA-1 and ApoC-III had the highest ROC AUC scores in the closure group indicating good to excellent classification power between the baseline and 3–12 month follow up samples, indicating a significant difference. Abundance ratios (baseline/follow up) for these proteins were also higher in the PFO closure sample set than in the no closure sample set, with the ApoC-III B/FU abundance ratio almost 2× higher in the closure sample group. In the no closure group, kininogen had the most significant ROC AUC score and its abundance ratio was ca 1.6× higher than in the closure group.

**Table 3:** **Abundance ratios and ROC AUC of albumin-bound proteins common to both PFO Closure and No Closure stroke samples**

GI	Protein	ROC AUC baseline Closure/Follow up	ROC AUC baseline No Closure/Follow up	Ratio baseline Closure/Follow up	Ratio baseline No Closure/Follow up
4557321	apolipoprotein A-I preproprotein [Homo sapiens]	0.950	0.76	1.46	1.26
4557323	apolipoprotein C-III precursor [Homo sapiens]	0.89	0.76	2.00	1.17
19923106	serum paraoxonase/arylesterase 1 precursor [Homo sapiens]	0.87	0.80	1.69	1.26
4502157	apolipoprotein C-I precursor [Homo sapiens]	0.87	0.52*	0.92	0.9
32483410	vitamin D-binding protein isoform 1 precursor [Homo sapiens]	0.87	0.80	2.16	1.53
32130518	apolipoprotein C-II precursor [Homo sapiens]	0.84	0.80	0.92	1.35
4504893	kininogen-1 isoform 2 precursor [Homo sapiens]	0.655*	0.92	0.85	1.35
4502149	apolipoprotein A-II preproprotein [Homo sapiens]	0.628*	0.684*	0.92	1.08

*poor classification.

### IPA Downstream analysis of albumin-bound proteins

**Figure 3:**
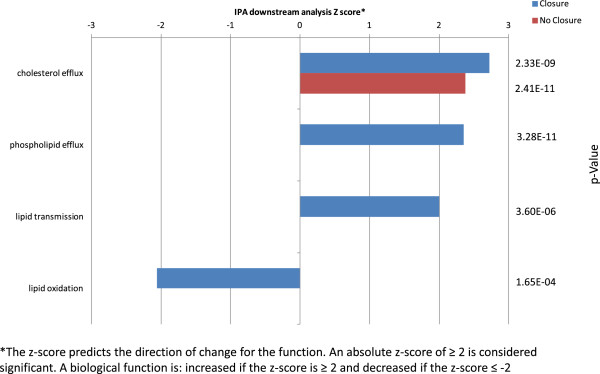
**IPA downstream analysis of differently abundant albumin-bound proteins in PFO closure** ***vs*** **no-closure sample sets.** The IPA biological function downstream analysis is based on z-score and p-value. In our analyses, the z-score values indicated that a function was expected to be increased (positive z-score) or decreased (negative z-score) in PFO closure relative to no-closure samples.

The protein datasets were analyzed by IPA Downstream Analysis. The IPA biological downstream analysis is based on z-score and p-value (see Methods). Biological functions defined in the IPA knowledge database that are expected to be increased or decreased according to the protein abundance in the dataset are identified by the z-score algorithm and the score reflects the significance of the prediction (the Z-scores do not measure the amount of increase or decrease of a function). In our analyses, the z-score values indicated that a function was expected to be increased (positive z-score) or decreased (negative z-score) in PFO closure relative to no-closure samples. The probability that the association between the proteins in our datasets and a related biological function defined in the IPA knowledge database was considered significant if the p-value was ≤0.01 as determined by using Fisher’s exact *T* test. The results from the IPA downstream analysis predicted an increase (z-score >2.0) in PFO closure *vs* no-closure in several functional categories. The most significant function was cholesterol efflux, with p-values of 2.33E-09, 2.41E-11 in the PFO closure and no-closure data sets, respectively and z-scores of 2.73 and 2.37 in the PFO closure and no-closure data sets, respectively (Figure 3). Conversely, the analysis predicted a decrease in lipid oxidation with a z-score of −2.06 and a p-value 1.65E-04 in PFO closure samples. There was no significant value for decreased lipid oxidation in the no-closure samples (Figure 3). This analysis suggests increased cholesterol and phospholipid efflux and decreased lipid oxidation in the PFO closure sample group as compared to the no closure sample group.

### Total cholesterol and HDL levels measured by colorimetric assay

**Figure 4:**
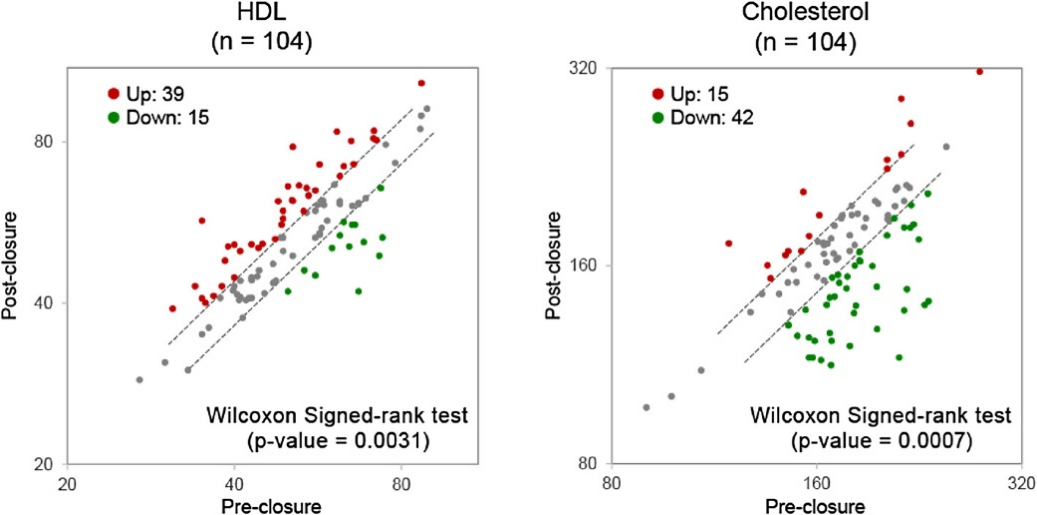
**HDL and Cholesterol levels measured by colorimetric assay in samples before and after stroke–related PFO closure.** HDL and cholesterol were measured in a separate validation cohort (n = 104, Table 1). All patients had CLIO certified cholesterol and HDL measured pre and post PFO closure at the same time interval as the exploratory cohort. Conditional logistic regression analysis (Table 4) demonstrated that cholesterol levels were statistically decreased (p-value 0.0007) and HDL levels were statistically increased (p-value 0.0031) post PFO closure when compared to baseline levels.

In order to test the predictions for increased cholesterol efflux and decreased lipid oxidation resulting from the IPA analysis described above, we validated total **c**holesterol and HDL levels in a separate and larger cohort (n = 104) of PFO closure patient samples (Table 1) using a standard commercial colorimetric assay (Figure 4, Table 4). The results from these measurements were consistent with the IPA predictions. Cholesterol levels were statistically decreased (p-value 0.0007) and conversely, HDL levels were statistically increased (p-value 0.0031) post PFO closure compared to baseline levels. We measured HDL in the samples since in addition to its cardioprotective and anti-arthrogenic effects, HDL has significant antioxidant properties [27].

**Table 4:** **Conditional logistic regression analysis**

Conditional logistic regression analysis			
Variables	B	Odd ratio (95% confidence interval)	P-value
HDL	−0.0567	0.9449 (0.8973, 0.9950)	0.0315
CHOL	0.0214	1.0217 (1.0055, 1.0381)	0.0085
Lipid-lowing Agent	−0.8454	0.4294 (0.0850, 2.1695)	0.3064

In the validation cohort, after adjusting for confounders such as lipid lowering agent, HDL and cholesterol remain (statistically significantly) changed post-PFO closure.

## Discussion and conclusions

The anatomy of PFO suggests that, in addition to thrombi, it can also allow potentially harmful circulatory factors to travel directly from the venous to the arterial circulation, a concept important in finding novel therapeutic targets for PFO-related neurovascular injury [1].

In an effort to unravel biological changes and discover potential new biomarkers related to PFO endovascular closure and stroke, we applied immuno-enrichment and high resolution LC-MS/MS to investigate the albumin-bound proteins in a small cohort of PFO stroke patients with and without endovascular closure at baseline and 3–12 month follow up.

One of the recurring problems with immunoenrichment for protein-protein interaction studies has been non-specific interactions with capture antibodies, binding matrices or both [25]. In order to minimize non-specific interactions, we used MSIA (mass spectrometric immunoassay) technology which has been demonstrated to minimize non-specific binding [25, 28, 29]. In addition, we developed a negative control with an antibody to a non-human protein and thus were able to identify and exclude non-specific interactions.

Our results demonstrated that the MS profiles of albumin-bound proteins samples from PFO closure *vs* no-closure were strikingly different, with only 8 highly abundant proteins typically found in HDL common to both sample sets. Biological analysis of the complete protein data sets using IPA downstream analysis suggested that PFO closure was statistically significantly associated with increased cholesterol efflux and decreased lipid oxidation. The protein abundance data also showed that albumin-bound ApoA-I and several other proteins were significantly increased (1.2-1.7 fold) and also had ROC AUC scores (>0.8) in PFO closure (*vs* no-closure) donor samples indicating high classification power. To explore these results further in a separate and larger cohort of PFO stroke patients, HDL and cholesterol were measured using standard clinical methods. These data showed that HDL was statistically significantly increased and cholesterol was statistically significantly decreased following endovascular closure.

ApoA-I is the most abundant protein in HDL and the HDL-mediated ApoA-I pathway and passive diffusion are the only well described pathways for cholesterol efflux [17–19]. However, recent studies [20, 21] have shown that albumin mediates cholesterol efflux in cultured fibroblasts and endothelial cells but the mechanism is not known. A study in rats demonstrated that albumin played a major role in cholesterol transport in circulation, with *ca* 24% of the nonesterified cholesterol bound to albumin [30]. Removal of albumin from plasma by immunoaffinity chromatography reduced total efflux in cultured fibroblasts by as much as 40% [31] and in a recent study using cultured mouse macrophage cells, the removal of albumin from plasma resulted in a significant reduction of cholesterol efflux [21]. The researchers in the most recent study proposed that albumin may act as a shuttle to enhance the aqueous diffusion of free cholesterol and not take part in a receptor-mediated process. However, our and others [9] observation that a significant amount of ApoA-I is bound to albumin may suggest why albumin is effective in promoting cholesterol efflux. Since lipid-poor ApoA-I acts as the cholesterol acceptor in the ABCA1pathway [32], we propose that albumin could be acting as a shuttle to deliver lipid-poor ApoA-1 to the cell surface, thereby enhancing ABCA1 mediated as well as passive cholesterol efflux. A recent publication demonstrated that a high percentage of Apo A-1 recovered from human atheroma is oxidized by myeloperoxidase and dysfunctional with respect to cholesterol efflux [33]. Apo A-1 bound to albumin may be unavailable to oxidation by myeloperoxidase and therefore the high concentration of albumin (>50% of the total protein) and, as a result, albumin-bound ApoA-I in human plasma suggests that this proposed mechanism could be a significant factor in total cholesterol binding and efflux. *In vivo* experiments have demonstrated that analbuminemic patients had decreased ApoA-I and increased free cholesterol (and ApoB) [34]. The reported, but controversial, protective effects of albumin in cardiovascular and cerebrovascular disease [35] may be explained by the role of albumin in cholesterol efflux. Whereas little is known about the equilibrium of albumin-bound and HDL-associated ApoA-I, our preliminary findings are provocative in suggesting that PFO repair may alter the circulatory composition in stroke patients and potentially improve the cholesterol profile.

Numerous publications have demonstrated that albumin can be a rich source of potential biomarkers as well as having pleiotropic functions including lipid, drug and metabolite transport and anti-oxidation [6–8, 11–14]. In our dataset, we identified a collection of low-abundance proteins and potential biomarkers. Low abundance proteins detected in the albumin-bound fraction of PFO closure samples (Additional file 4) included receptors (integrin alpha-7 isoform 2 precursor), transcription factors (HMG box-containing protein 1 isoform 2) and an acylpeptide hydrolase (acylamino-acid-releasing enzyme), that can play an important role in destroying oxidatively damaged proteins in living cells [36]. This finding supports the hypothesis that low-abundance biomarkers circulating in blood may exist bound to albumin, preventing their clearance by the kidneys [12].

Proteins with significantly increased abundance ratios in PFO closure *vs* no-closure samples included PON 1, another key component of HDL and partially responsible for its antioxidant and atheroprotective properties [37], and vitamin D-binding protein. Proteins with decreased abundance ratios in PFO closure *vs* no-closure samples included kininogen and Apo C-II.

The list of highly abundant albumin-bound proteins identified in our study is consistent with albumin-bound proteins identified in previous studies [9, 16].

The collective data presented in this study demonstrate that analysis of albumin-bound proteins could provide a valuable tool for biomarker discovery on the effects of PFO endovascular closure. In addition, the results suggest that PFO endovascular closure can potentially have effects on HDL, cholesterol and albumin-bound ApoA-I abundance, therefore possibly providing benefits in cardioprotective functions. The potential benefit of raising HDL levels has been discussed but remains controversial [38]. However, most experimental studies have looked at over or under- expression of single genes and it is clear that a better understanding of the mechanisms of action and multiple effects of HDL are needed [39, 40].

These findings in this study are provocative, however, further studies with multiple potential confounders such as exercise, genetic factors, alcohol, hormonal influences, and nutrition status (which all affect albumin and lipid profiles) will be carried out in larger patient cohorts in future studies.

## Electronic supplementary material

Additional file 1: **Search report summary of file.** (DOCX 18 KB)

Additional file 2: **Proteins and peptide sequences.** (XLSX 603 KB)

Additional file 3: **Annotation.** (XLSX 91 KB)

Additional file 4: Table S3: Abundance ratios and Protein ID’s for all albumin-bound proteins in both PFO Closure and No Closure stroke samples. (XLSX 16 KB)

## Abbreviations

Ab: Antibody
ACN: Acetonitrile
Apo: Apolipoprotein
AUC: Area under the curve
B: Baseline
BL: Betalactoglobulin
CTA: Computed tomography angiogram
CT: Computed tomography
CaCl2: Calcium chloride
EDTA: Ethylenediaminetetraacetic acid
ESI: Electrospray ionization
FU: 3–12 month follow up
GPCR: G protein coupled receptor
HDL: High density lipoprotein
IRB: Institutional review board
LC: Liquid chromatography
IPA: Ingenuity pathways analysis
MS: Mass spectrometry
MS/MS: Tandem mass spectrometry
*m/z*: Mass to charge ratio
MSIA: Mass spectrometric immunoassay
MRI: Magnetic resonance imaging
MRA: Magnetic resonance angiogram
PBS: Phosphate buffered saline
PFO: Patent foramen ovale
PTH: Parathyroid hormone
PTHR: PTH parathyroid hormone receptor
RPM: Revolutions per minute
ROC: Receiver operating characteristic.
