# Supplementary material for: Proteomic signatures of serum albumin-bound proteins from stroke patients with and without endovascular closure of PFO are significantly different and suggest a novel mechanism for cholesterol efflux
Source: Clin Proteomics. 2015 Jan 13;12(1):2. doi: 10.1186/1559-0275-12-2 (PMC4305391; doi:10.1186/1559-0275-12-2)
Supplement: Supplementary file 1 — Additional file 1: Search report summary of file. (DOCX 18 KB) [file 12014_2014_87_MOESM1_ESM.docx]

Additional file 1.

Search report summary of file(s):

- P:\VelosOrbi-Pro\NingAlbuminomics0628-2013\Albumin\LowRes_Sequest_PDv1.4\NingAlbuminomicsPlate1-Albumin-Sample-28.msf

Created with Discoverer version: 1.4.0.288

================================================================================

Number of filtered/unfiltered result items:

- 70/71 protein group(s)

- 178/178 merged protein(s)

- 283/283 peptide(s)

- 421/421 PSM(s)

- 7264/7264 search input(s)

================================================================================

Peptide Grouping Options

- Show peptide groups: True

- Group peptides by: Mass and Sequence

Protein Grouping Options

- Enable protein grouping: True

- Consider leucine and isoleucine as equal: True

- Consider only PSMs with confidence at least: Medium

- Consider only PSMs with delta Cn better than: 0.15

- Apply strict maximum parsimony principle: True

Data reduction filters:

- Peptide Confidence (Minimum confidence: High)

No result filters applied

================================================================================

Summary of file P:\VelosOrbi-Pro\NingAlbuminomics0628-2013\Albumin\LowRes_Sequest_PDv1.4\NingAlbuminomicsPlate1-Albumin-Sample-28.msf

Workflow created with Discoverer version: 1.4.0.288 (DBVersion:79)

================================================================================

Search name: NingAlbuminomicsPlate1-Albumin-Sample-28

Search description: -

Search date: 04/07/2014 11:28:46

================================================================================

The pipeline tree:

------------------

|-(0) Spectrum Files

|-(7) Spectrum Selector

|-(8) SEQUEST

|-(9) Percolator

|-(4) Annotation

------------------------------------------------------------------------------

Processing node 0: Spectrum Files

------------------------------------------------------------------------------

Input Data:

-----------------------------

File Name(s): X:\LabData\VelosOrbi-Pro\NingAlbuminomics0628-2013\NingAlbuminomicsPlate1-Albumin-Sample-28.raw

------------------------------------------------------------------------------

Processing node 7: Spectrum Selector

------------------------------------------------------------------------------

1. General Settings:

-----------------------------

Precursor Selection: Use MS1 Precursor

Use New Precursor Reevaluation: True

2. Spectrum Properties Filter:

-----------------------------

Lower RT Limit: 0

Upper RT Limit: 0

First Scan: 0

Last Scan: 0

Lowest Charge State: 0

Highest Charge State: 0

Min. Precursor Mass: 350 Da

Max. Precursor Mass: 5000 Da

Total Intensity Threshold: 0

Minimum Peak Count: 1

3. Scan Event Filters:

-----------------------------

MS Order: Is MS2

Min. Collision Energy: 0

Max. Collision Energy: 1000

Scan Type: Is Full

4. Peak Filters:

-----------------------------

S/N Threshold (FT-only): 1.5

5. Replacements for Unrecognized Properties:

-----------------------------

Unrecognized Charge Replacements: Automatic

Unrecognized Mass Analyzer Replacements: ITMS

Unrecognized MS Order Replacements: MS2

Unrecognized Activation Type Replacements: CID

Unrecognized Polarity Replacements: +

6. Just for Testing:

-----------------------------

Precursor Clipping Range Before: 2.5 Da

Precursor Clipping Range After: 5.5 Da

------------------------------------------------------------------------------

Processing node 8: SEQUEST

------------------------------------------------------------------------------

1. Input Data:

-----------------------------

Protein Database: 040212-RefSeqHuman.fasta

Enzyme Name: Trypsin (Full)

Maximum Missed Cleavage Sites: 1

1.1 Peptide Scoring Options:

-----------------------------

Maximum Peptides Considered: 500

Maximum Peptides Output: 10

Calculate Probability Scores: False

Absolute XCorr Threshold: 0.4

Fragment Ion Cutoff Percentage: 0.1

Peptide Without Protein XCorr Threshold: 1.5

1.2 Protein Scoring Options:

-----------------------------

Maximum Protein References Per Peptide: 100

Protein Relevance Threshold: 1.5

Peptide Relevance Factor: 0.4

2. Tolerances:

-----------------------------

Precursor Mass Tolerance: 5 ppm

Fragment Mass Tolerance: 0.25 Da

Use Average Precursor Mass: False

Use Average Fragment Masses: False

3. Ion Series:

-----------------------------

Use Neutral Loss a Ions: True

Use Neutral Loss b Ions: True

Use Neutral Loss y Ions: True

Weight of a Ions: 0

Weight of b Ions: 1

Weight of c Ions: 0

Weight of x Ions: 0

Weight of y Ions: 1

Weight of z Ions: 0

4. Dynamic Modifications:

-----------------------------

Max. Modifications Per Peptide: 4

1. Dynamic Modification: Oxidation / +15.995 Da (M)

5. Static Modifications:

-----------------------------

1. Static Modification: Carboxymethyl / +58.005 Da (C)

------------------------------------------------------------------------------

Processing node 9: Percolator

------------------------------------------------------------------------------

1. Input Data:

-----------------------------

Maximum Delta Cn: 0.05

2. Decoy Database Search:

-----------------------------

Target FDR (Strict): 0.01

Target FDR (Relaxed): 0.05

Validation based on: q-Value

------------------------------------------------------------------------------

Processing node 4: Annotation

------------------------------------------------------------------------------

================================================================================

Further information:

SEQUEST (8):

- Number of sequences searched: 29969

Percolator (9):

- Percolator Output: Results for SEQUEST (8):

Iteration 1 : After the iteration step, 519 target PSMs with q<0.01 were estimated by cross validation

Iteration 2 : After the iteration step, 532 target PSMs with q<0.01 were estimated by cross validation

Iteration 3 : After the iteration step, 537 target PSMs with q<0.01 were estimated by cross validation

Iteration 4 : After the iteration step, 542 target PSMs with q<0.01 were estimated by cross validation

Iteration 5 : After the iteration step, 544 target PSMs with q<0.01 were estimated by cross validation

Iteration 6 : After the iteration step, 546 target PSMs with q<0.01 were estimated by cross validation

Iteration 7 : After the iteration step, 548 target PSMs with q<0.01 were estimated by cross validation

Iteration 8 : After the iteration step, 549 target PSMs with q<0.01 were estimated by cross validation

Iteration 9 : After the iteration step, 549 target PSMs with q<0.01 were estimated by cross validation

Iteration 10 : After the iteration step, 549 target PSMs with q<0.01 were estimated by cross validation

Feature name Raw weight Normalized weight

XCorr 0.1136 0.0825

SpScore 0.0028 0.5407

Delta Cn From Second PSM 1.5071 0.5625

Binomial Score 0.1198 5.1745

Isolation Interference [%] 0.0299 0.6057

MH+ [Da] -0.0002 -0.1031

Delta Mass [Da] 337.1399 1.2172

Delta Mass [ppm] -0.3197 -0.8497

Absolute Delta Mass [Da] -409.5748 -0.9205

Absolute Delta Mass [ppm] 0.7553 1.0912

Peptide Length 0.1807 0.8135

Is z=1 1.0374 0.472

Is z=2 -2.3035 -1.1247

Is z=3 3.1863 0.9277

Is z=4 10.771 0.8148

Is z=5 7.3502 0.1394

Is z>5 0 0

# Missed Cleavages -0.0378 -0.0188

Log Peptides Matched 0 0

Log Total Intensity 1.9507 0.9499

Fraction Matched Intensity [%] 0.0417 0.4338

Fragment Coverage Series A, B, C [%] -0.0157 -0.3122

Fragment Coverage Series X, Y, Z [%] 0.0387 0.7959

Log Matched Fragment Series Intensities A, B, C -0.1027 -0.1599

Log Matched Fragment Series Intensities X, Y, Z -1.1461 -1.8086

Longest Sequence Series A, B, C -0.0983 -0.1552

Longest Sequence Series X, Y, Z -0.2296 -0.4261

IQR Fragment Delta Mass [Da] 4.3737 0.2398

IQR Fragment Delta Mass [ppm] -0.0062 -0.7281

Mean Fragment Delta Mass [Da] -8.6347 -0.4463

Mean Fragment Delta Mass [ppm] -0.0012 -0.1756

Mean Absolute Fragment Delta Mass [Da] -23.3683 -0.7418

Mean Absolute Fragment Delta Mass [ppm] 0.0101 1.1156

m0 -21.154 -7.3476

================================================================================

Processing details:

04/07/2014 11:35 AM (8):SEQUEST: Total search time was 2 min 44 s.

04/07/2014 11:35 AM (4):Annotation: Annotation: Total 1685 of 1685 proteins found ...

04/07/2014 11:35 AM (4):Annotation: Store annotations: 1685 of 1685 proteins found ...

04/07/2014 11:35 AM (4):Annotation: There is a newer version of protein annotations available: updating AnnotationsDB...

04/07/2014 11:35 AM (4):Annotation: ProteinCenter data release = 1395767267000, ProteinCenter data version = 267

04/07/2014 11:35 AM (4):Annotation: Retrieving annotations from ProteinCenter (http://webservice.proteincenter.proxeon.com/ProXweb/)...

04/07/2014 11:35 AM (9):Percolator: Performing percolator for SEQUEST (8) took 1 min 44 s.

04/07/2014 11:35 AM (8):SEQUEST: Search completed

04/07/2014 11:35 AM (8):SEQUEST: 1685 protein(s) + 1534 decoy proteins scored and inserted into result file in 6.7 s.

04/07/2014 11:35 AM (8):SEQUEST: 1685 protein(s) scored

04/07/2014 11:35 AM (8):SEQUEST: Search result finalization started.

04/07/2014 11:35 AM (9):Percolator: Start reading Percolator results

04/07/2014 11:35 AM (9):Percolator: Processing took 9.204 cpu seconds or 9 seconds wall time

04/07/2014 11:35 AM (9):Percolator: Calibrating statistics - calculating Posterior error probabilities (PEPs)

04/07/2014 11:35 AM (9):Percolator: PSMId score q-value posterior_error_prob peptide proteinIds

04/07/2014 11:35 AM (9):Percolator: New pi_0 estimate on merged list gives 286 peptides over q=0.0100

04/07/2014 11:35 AM (9):Percolator: Calibrating statistics - calculating q values

04/07/2014 11:35 AM (9):Percolator: Selecting pi_0=0.8754

04/07/2014 11:35 AM (9):Percolator: Tossing out "redundant" PSMs keeping only the best scoring PSM for each unique peptide.

04/07/2014 11:34 AM (9):Percolator: Merging results from 3 datasets

04/07/2014 11:34 AM (9):Percolator: Selected feature number 4 as initial search direction, could separate 257 positives in that direction

04/07/2014 11:34 AM (9):Percolator: Selected feature number 4 as initial search direction, could separate 312 positives in that direction

04/07/2014 11:34 AM (9):Percolator: Selected feature number 4 as initial search direction, could separate 280 positives in that direction

04/07/2014 11:34 AM (9):Percolator: Reseting score vector, using default vector

04/07/2014 11:34 AM (9):Percolator: Less identifications (422 vs 424) after percolator processing than before processing

04/07/2014 11:34 AM (9):Percolator: Found 422 target PSMs scoring over 1.0000% FDR level on testset

04/07/2014 11:34 AM (9):Percolator: 0.1136 0.0028 1.5071 0.1198 0.0299 -0.0002 337.1399 -0.3197 -409.5748 0.7553 0.1807 1.0374 -2.3035 3.1863 10.7710 7.3502 0.0000 -0.0378 0.0000 1.9507 0.0417 -0.0157 0.0387 -0.1027 -1.1461 -0.0983 -0.2296 4.3737 -0.0062 -8.6347 -0.0012 -23.3683 0.0101 -21.1540

04/07/2014 11:34 AM (9):Percolator: 0.0825 0.5407 0.5625 5.1745 0.6057 -0.1031 1.2172 -0.8497 -0.9205 1.0912 0.8135 0.4720 -1.1247 0.9277 0.8148 0.1394 0.0000 -0.0188 0.0000 0.9499 0.4338 -0.3122 0.7959 -0.1599 -1.8086 -0.1552 -0.4261 0.2398 -0.7281 -0.4463 -0.1756 -0.7418 1.1156 -7.3476

04/07/2014 11:34 AM (9):Percolator: XCorr SpScore Delta Cn From Second PSM Binomial Score Isolation Interference [%] MH+ [Da] Delta Mass [Da] Delta Mass [ppm] Absolute Delta Mass [Da] Absolute Delta Mass [ppm] Peptide Length Is z=1 Is z=2 Is z=3 Is z=4 Is z=5 Is z>5 # Missed Cleavages Log Peptides Matched Log Total Intensity Fraction Matched Intensity [%] Fragment Coverage Series A, B, C [%] Fragment Coverage Series X, Y, Z [%] Log Matched Fragment Series Intensities A, B, C Log Matched Fragment Series Intensities X, Y, Z Longest Sequence Series A, B, C Longest Sequence Series X, Y, Z IQR Fragment Delta Mass [Da] IQR Fragment Delta Mass [ppm] Mean Fragment Delta Mass [Da] Mean Fragment Delta Mass [ppm] Mean Absolute Fragment Delta Mass [Da] Mean Absolute Fragment Delta Mass [ppm] m0

04/07/2014 11:34 AM (9):Percolator: # first line contains normalized weights, second line the raw weights

04/07/2014 11:34 AM (9):Percolator: Obtained weights (only showing weights of first cross validation set)

04/07/2014 11:34 AM (9):Percolator: Iteration 10 : After the iteration step, 549 target PSMs with q<0.01 were estimated by cross validation

04/07/2014 11:34 AM (9):Percolator: Iteration 9 : After the iteration step, 549 target PSMs with q<0.01 were estimated by cross validation

04/07/2014 11:34 AM (9):Percolator: Iteration 8 : After the iteration step, 549 target PSMs with q<0.01 were estimated by cross validation

04/07/2014 11:34 AM (9):Percolator: Iteration 7 : After the iteration step, 548 target PSMs with q<0.01 were estimated by cross validation

04/07/2014 11:34 AM (9):Percolator: Iteration 6 : After the iteration step, 546 target PSMs with q<0.01 were estimated by cross validation

04/07/2014 11:34 AM (9):Percolator: Iteration 5 : After the iteration step, 544 target PSMs with q<0.01 were estimated by cross validation

04/07/2014 11:34 AM (9):Percolator: Iteration 4 : After the iteration step, 542 target PSMs with q<0.01 were estimated by cross validation

04/07/2014 11:34 AM (9):Percolator: Iteration 3 : After the iteration step, 537 target PSMs with q<0.01 were estimated by cross validation

04/07/2014 11:34 AM (9):Percolator: Iteration 2 : After the iteration step, 532 target PSMs with q<0.01 were estimated by cross validation

04/07/2014 11:34 AM (9):Percolator: Iteration 1 : After the iteration step, 519 target PSMs with q<0.01 were estimated by cross validation

04/07/2014 11:34 AM (9):Percolator: ---Training with Cpos selected by cross validation, Cneg selected by cross validation, fdr=0.01

04/07/2014 11:34 AM (9):Percolator: Reading in data and feature calculation took 8.524 cpu seconds or 9 seconds wall time

04/07/2014 11:34 AM (9):Percolator: Estimating 424 over q=0.01 in initial direction

04/07/2014 11:34 AM (9):Percolator: Selected feature number 4 as initial search direction, could separate 257 positives in that direction

04/07/2014 11:34 AM (9):Percolator: Selected feature number 4 as initial search direction, could separate 312 positives in that direction

04/07/2014 11:34 AM (9):Percolator: Selected feature number 4 as initial search direction, could separate 280 positives in that direction

04/07/2014 11:34 AM (9):Percolator: selecting cneg by cross validation

04/07/2014 11:34 AM (9):Percolator: selecting cpos by cross validation

04/07/2014 11:34 AM (9):Percolator: Train/test set contains 4174 positives and 4166 negatives, size ratio=1.00192 and pi0=1

04/07/2014 11:34 AM (9):Percolator: 31e77142-29e9-402c-9ec2-8468a9513af0 e39a792e-622c-452d-b49b-59809cad79d0 Delta Cn From Second PSM Binomial Score b8754504-e95e-476b-b9a4-454d4bb53aeb 1d91a87b-953a-4887-9f22-f75a497a3538 Delta Mass [Da] Delta Mass [ppm] Absolute Delta Mass [Da] Absolute Delta Mass [ppm] Peptide Length Is z=1 Is z=2 Is z=3 Is z=4 Is z=5 Is z>5 041eb6d5-e486-44a0-9bc1-19e25811c686 Log Peptides Matched Log Total Intensity Fraction Matched Intensity [%] Fragment Coverage Series A, B, C [%] Fragment Coverage Series X, Y, Z [%] Log Matched Fragment Series Intensities A, B, C Log Matched Fragment Series Intensities X, Y, Z Longest Sequence Series A, B, C Longest Sequence Series X, Y, Z IQR Fragment Delta Mass [Da] IQR Fragment Delta Mass [ppm] Mean Fragment Delta Mass [Da] Mean Fragment Delta Mass [ppm] Mean Absolute Fragment Delta Mass [Da] Mean Absolute Fragment Delta Mass [ppm]

04/07/2014 11:34 AM (9):Percolator: Features:

04/07/2014 11:34 AM (9):Percolator: enzyme=Trypsin

04/07/2014 11:34 AM (9):Percolator: Hyperparameters fdr=0.01, Cpos=0, Cneg=0, maxNiter=10

04/07/2014 11:34 AM (9):Percolator: Started Mon Apr 07 11:34:46 2014

04/07/2014 11:34 AM (9):Percolator: C:\Program Files\Thermo\Discoverer 1.4\Tools\Percolator\percolator.exe -X C:\ProgramData\Thermo\Discoverer 1.4\Scratch\7724a8e0-dd75-445f-8bff-e3abdb559d88\output.xml -Z C:\ProgramData\Thermo\Discoverer 1.4\Scratch\7724a8e0-dd75-445f-8bff-e3abdb559d88\input.xml

04/07/2014 11:34 AM (9):Percolator: Issued command:

04/07/2014 11:34 AM (9):Percolator: Department of Genome Sciences at the University of Washington.

04/07/2014 11:34 AM (9):Percolator: Written by Lukas K├ñll (lukall@u.washington.edu) in the

04/07/2014 11:34 AM (9):Percolator: Copyright (c) 2006-9 University of Washington. All rights reserved.

04/07/2014 11:34 AM (9):Percolator: Percolator version 2.04, Build Date Feb 1 2012 03:35:34

04/07/2014 11:34 AM (9):Percolator: Starting Percolator

04/07/2014 11:34 AM (9):Percolator: The input file contains 4174 peptides, 4166 decoy peptides and 33 features.

04/07/2014 11:34 AM (9):Percolator: Creating input file for SEQUEST (8) took 1 min 19 s.

04/07/2014 11:33 AM (9):Percolator: Start calculating features for peptides of SEQUEST (8)

04/07/2014 11:33 AM (8):SEQUEST: Sending 264 decoy peptide hits (186 peptides) to result file

04/07/2014 11:33 AM (8):SEQUEST: Starting SEQUEST decoy search

04/07/2014 11:33 AM (8):SEQUEST: Sending 264 peptide hits (172 peptides) to result file

04/07/2014 11:33 AM (8):SEQUEST: Starting SEQUEST (search spectra 7000 - 7264)

04/07/2014 11:33 AM (8):SEQUEST: Sending 1000 decoy peptide hits (1112 peptides) to result file

04/07/2014 11:33 AM (8):SEQUEST: Starting SEQUEST decoy search

04/07/2014 11:33 AM (8):SEQUEST: Sending 1000 peptide hits (1150 peptides) to result file

04/07/2014 11:32 AM (8):SEQUEST: Starting SEQUEST (search spectra 6000 - 7000)

04/07/2014 11:32 AM (8):SEQUEST: Sending 1000 decoy peptide hits (2584 peptides) to result file

04/07/2014 11:32 AM (8):SEQUEST: Starting SEQUEST decoy search

04/07/2014 11:32 AM (8):SEQUEST: Sending 1000 peptide hits (2552 peptides) to result file

04/07/2014 11:32 AM (8):SEQUEST: Starting SEQUEST (search spectra 5000 - 6000)

04/07/2014 11:32 AM (8):SEQUEST: Sending 1000 decoy peptide hits (4581 peptides) to result file

04/07/2014 11:31 AM (8):SEQUEST: Starting SEQUEST decoy search

04/07/2014 11:31 AM (8):SEQUEST: Sending 1000 peptide hits (4542 peptides) to result file

04/07/2014 11:31 AM (8):SEQUEST: Starting SEQUEST (search spectra 4000 - 5000)

04/07/2014 11:31 AM (8):SEQUEST: Sending 1000 decoy peptide hits (4389 peptides) to result file

04/07/2014 11:31 AM (8):SEQUEST: Starting SEQUEST decoy search

04/07/2014 11:31 AM (8):SEQUEST: Sending 1000 peptide hits (4507 peptides) to result file

04/07/2014 11:30 AM (8):SEQUEST: Starting SEQUEST (search spectra 3000 - 4000)

04/07/2014 11:30 AM (8):SEQUEST: Sending 1000 decoy peptide hits (4542 peptides) to result file

04/07/2014 11:30 AM (8):SEQUEST: Starting SEQUEST decoy search

04/07/2014 11:30 AM (8):SEQUEST: Sending 1000 peptide hits (4611 peptides) to result file

04/07/2014 11:30 AM (8):SEQUEST: Starting SEQUEST (search spectra 2000 - 3000)

04/07/2014 11:30 AM (8):SEQUEST: Sending 1000 decoy peptide hits (3308 peptides) to result file

04/07/2014 11:29 AM (8):SEQUEST: Starting SEQUEST decoy search

04/07/2014 11:29 AM (8):SEQUEST: Sending 1000 peptide hits (3339 peptides) to result file

04/07/2014 11:29 AM (8):SEQUEST: Starting SEQUEST (search spectra 1000 - 2000)

04/07/2014 11:29 AM (8):SEQUEST: Sending 1000 decoy peptide hits (1513 peptides) to result file

04/07/2014 11:29 AM (8):SEQUEST: Starting SEQUEST decoy search

04/07/2014 11:29 AM (8):SEQUEST: Sending 1000 peptide hits (1540 peptides) to result file

04/07/2014 11:29 AM (8):SEQUEST: Starting SEQUEST (search spectra 0 - 1000)

04/07/2014 11:29 AM (8):SEQUEST: There is already an adequate decoy FASTA index.

04/07/2014 11:29 AM (8):SEQUEST: Looking for existing decoy FASTA index.

04/07/2014 11:29 AM (8):SEQUEST: There is already an adequate target FASTA index.

04/07/2014 11:29 AM (8):SEQUEST: Looking for existing target FASTA index.

04/07/2014 11:28 AM (7):Spectrum Selector: Reading from File 1 of 1:X:\LabData\VelosOrbi-Pro\NingAlbuminomics0628-2013\NingAlbuminomicsPlate1-Albumin-Sample-28.raw (16040 spectra total)
